# Supplementary material for: Primer Choice and Xylem-Microbiome-Extraction Method Are Important Determinants in Assessing Xylem Bacterial Community in Olive Trees
Source: Plants (Basel). 2022 May 16;11(10):1320. doi: 10.3390/plants11101320 (PMC9144944; doi:10.3390/plants11101320)
Supplement: Supplementary file 1 [file plants-11-01320-s001.zip › Table S1.pdf]

Table S1. Primers sequences and target region of rRNA used in the metabarcoding analysis of olive xylem sap.

PCR	Primers Pairs	Primer Forward	Primer Reverse	Region	Size (pb)	References
PCR1	799F+1062R	AACMGGATTAGATACCCKG	CTCACRRCACGAGCTGAC	V5-V6	270	(Chelius and Triplett, 2001; Diskin et al., 2017)
PCR2	799F+1115R	AACMGGATTAGATACCCKG	AGGGTTGCGCTCGTTG	V5-V6	320	(Laforest-Lapointe et al., 2017)
PCR3	967F+1391R	CAACGCGAAGAACCTTACC	GACGGGCGGTGWGTRCA	V6- V8	430	(Callahan et al., 2016; Sogin et al., 2006; Walker and Pace, 2007)
PCR4	799F+1193R	AACMGGATTAGATACCCKG	ACGTCATCCCCACCTTCC	V5- V7	400	(Beckers et al., 2016; Bodenhausen et al., 2013; Walker and Pace, 2007)
