# Supplementary figures and images for: Primer Choice and Xylem-Microbiome-Extraction Method Are Important Determinants in Assessing Xylem Bacterial Community in Olive Trees
Source: Plants (Basel). 2022 May 16;11(10):1320. doi: 10.3390/plants11101320 (PMC9144944; doi:10.3390/plants11101320)

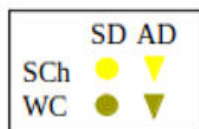

**PCR1 – 799F/1062R**

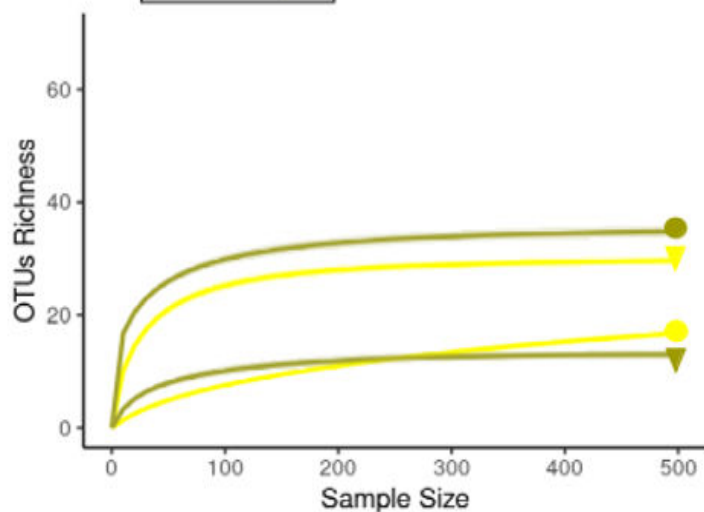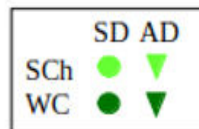

**PCR2 – 799F/1115R**

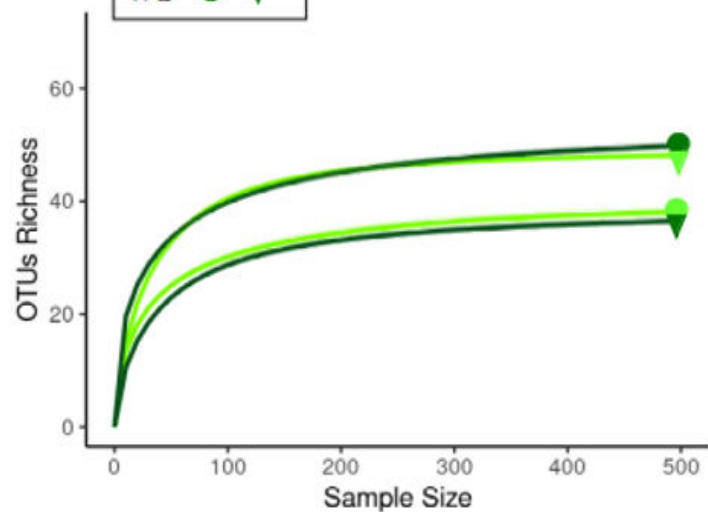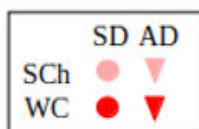

**PCR3 – 967F/1391R**

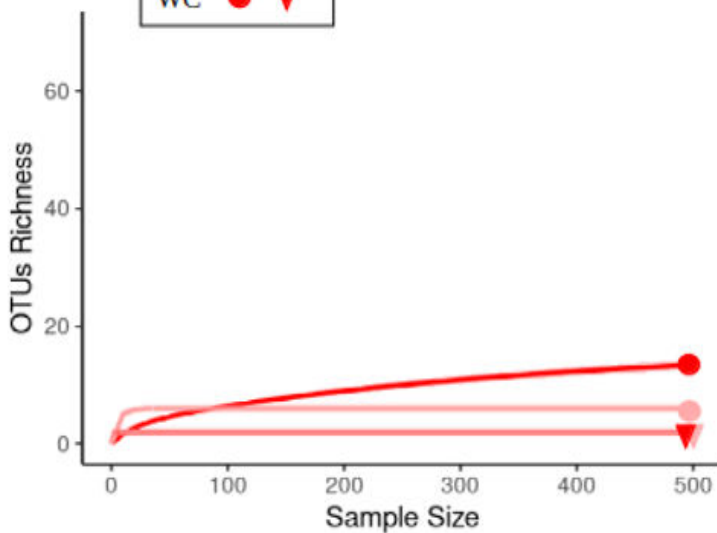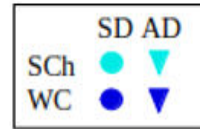

**PCR4 – 799F/1193R**

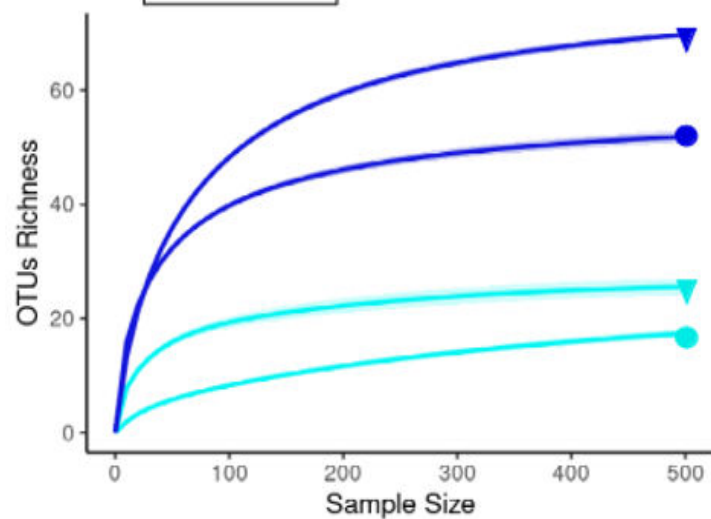

Supplement: Supplementary file 1 [file plants-11-01320-s001.zip › Figure S1.pdf]

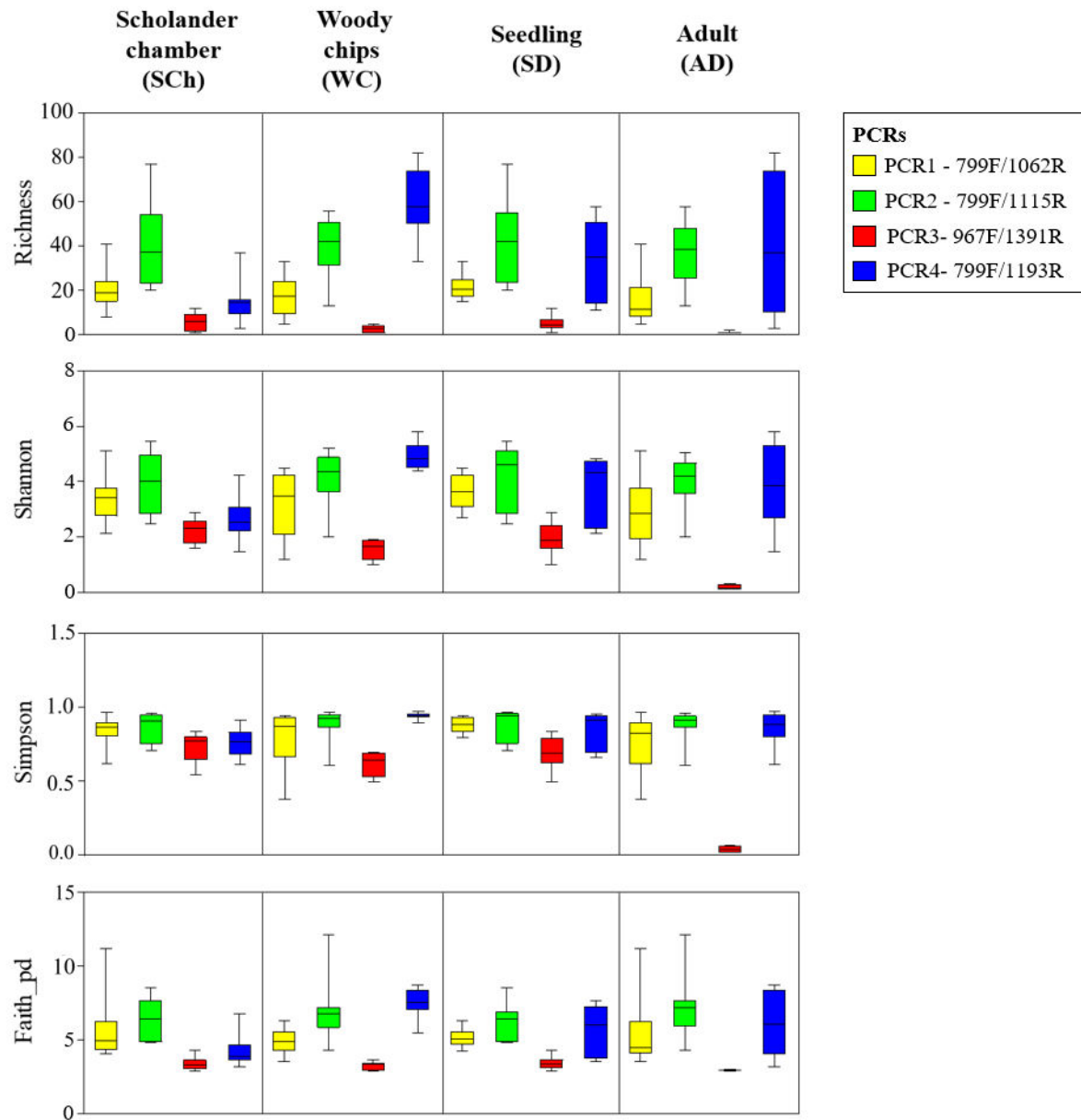

Supplement: Supplementary file 1 [file plants-11-01320-s001.zip › Figure S2.pdf]

## Scholander chamber (SCh)

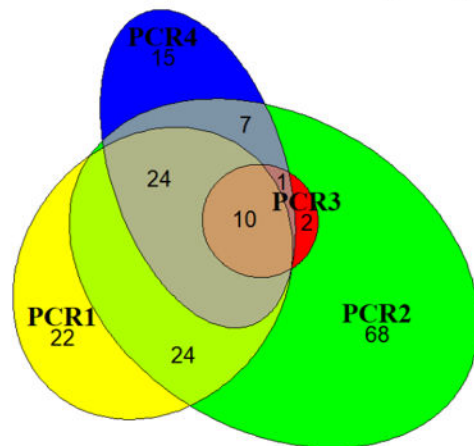

## Woody chips (WC)

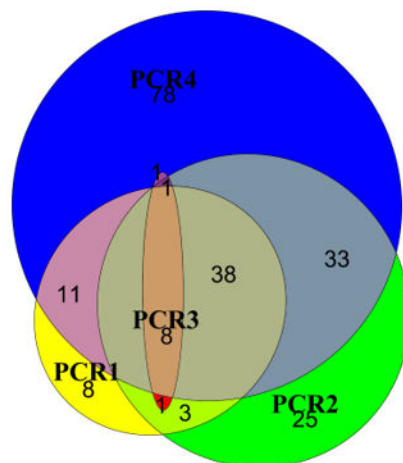

### PCRs

PCR1 - 799F/1062R

PCR2 - 799F/1115R

PCR3- 967F/1391R

PCR4- 799F/1193R

## Seedling (SD)

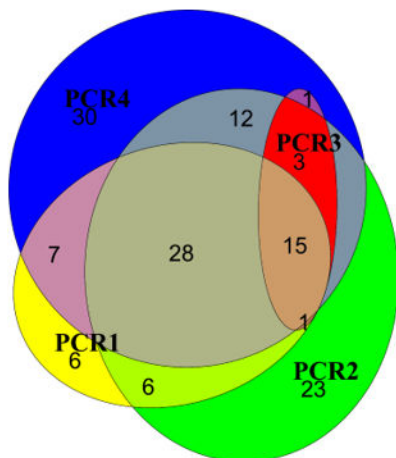

## Adult (AD)

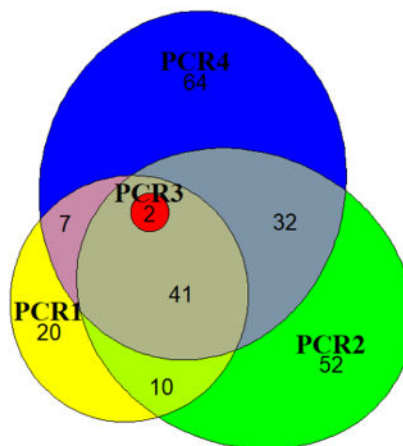

Supplement: Supplementary file 1 [file plants-11-01320-s001.zip › Figure S3.pdf]

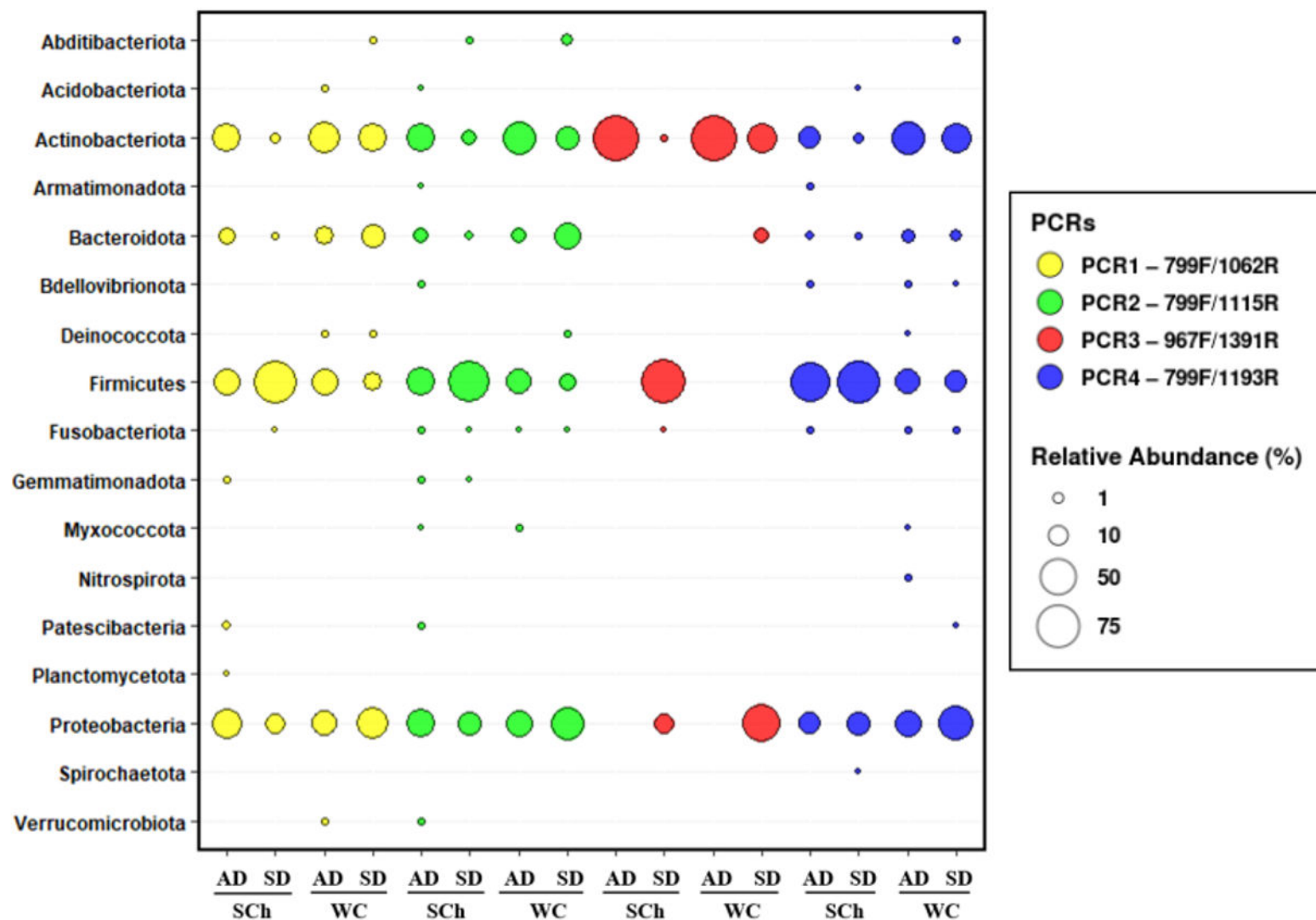

Supplement: Supplementary file 1 [file plants-11-01320-s001.zip › Figure S4.pdf]

## Scholander chamber

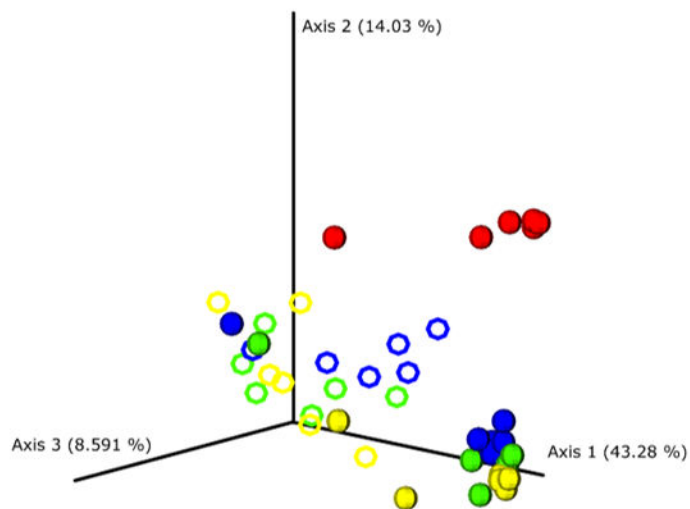

## Woody chips

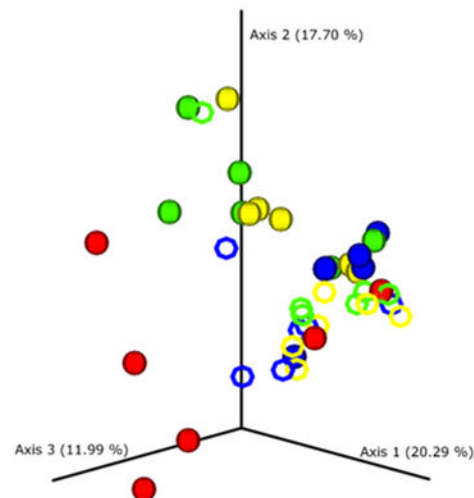

### PCRs

- PCR1 - 799F/1062R
- PCR2 - 799F/1115R
- PCR3- 967F/1391R
- PCR4- 799F/1193R

○ AD

● SD

## Seedling

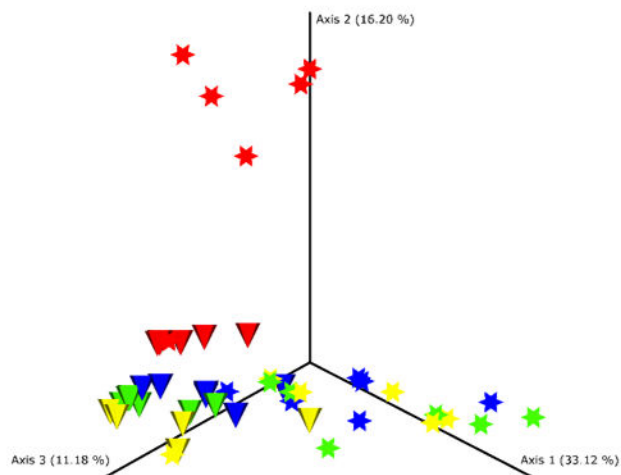

## Adult

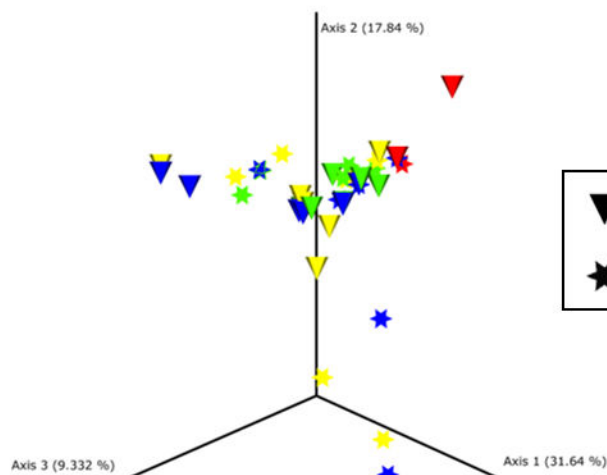

▼ Sch

★ WC

Supplement: Supplementary file 1 [file plants-11-01320-s001.zip › Figure S5.pdf]
